# The Health of Probationers

**Published:** 1887-10-29

**Authors:** 


### THE HEALTH OF PROBATIONERS.

Might I suggest that matrons would find a few simple printed rules of "precautions" of great service in keeping up the health standard of newly arrived probationers? The following instances will explain my meaning. Quite recently I heard of a probationer at one of the largest London hospitals losing her finger through blood-poisoning. She said that she had never once been warned of the need of care in dressing serious surgical cases, and that she must have had a slight abrasion on her finger. Another lady probationer, in what is considered an excellent training school for nurses, told me this week that she was nursing a case of typhoid, and that but for a few precautions, of which she had been told before she entered the hospital, she would not have known that any were necessary. In this very same hospital I was not surprised to hear that one of the probationers was suffering from typhoid ! I think matrons and sisters often fail to realise the extreme ignorance of probationers, and in consequence they do not warn them of the dangers of ordinary ward work. At the Brownlow Hill Infirmary an admirable little book used to be given to each probationer on her arrival. It was so small that it could be easily carried in the pocket, and the directions were few and very clear. They comprised the care needed in examining the hands each morning to see if there were scratches or abrasions of any kind, and if so the necessity of applying strapping, also the need for thorough cleansing of the hand after dressing a "bad case" ; the simple precautions needful in nursing typhoid, diphtheric, and ophthalmic cases, and some other rules for personal health and management which I do not now remember, but which were most practical and useful. These books were hand-printed by one of the patients, and were therefore of no expense ; but I have heard many of the probationers testify gratefully to the great service these rules had been to them. I think many poisoned fingers and "hospital sore-throats" might be avoided if probationers more fully realised the need of care and how to take it; they cannot be expected to know the dangers, and the sisters and nurses are often too busy to warn them. A little book such as I mention would, I think, be of the utmost use, dealing, as it would, exclusively with the personal health of the probationer. It is a pity that the general health should suffer, or serious accidents happen, such as blood-poisoning, when they might be avoided; and it is certainly the rank and file of probationers who suffer more in this way than the nurses and sisters, who, it may be supposed, have learnt to be careful through experience.—Faithfully yours, J. Wilson, Hon. Sec.

Workhouse Infirmary Nursing Association.

